# Supplementary material for: Highly Active Nanocrystalline ZnO and Its Photo-Oxidative Properties towards Acetone Vapor
Source: Micromachines (Basel). 2023 Apr 23;14(5):912. doi: 10.3390/mi14050912 (PMC10223819; doi:10.3390/mi14050912)
Supplement: Supplementary file 1 [file micromachines-14-00912-s001.zip › micromachines-2307722-supplementary.pdf]

Supplementary Information

for article

Highly Active Nanocrystalline ZnO and Its Photo-Oxidative Properties towards Acetone Vapor

Artem Chizhov [1,*], Pavel Kutukov [1], Alexander Gulin [2], Artyom Astafiev [2] and Marina Rumyantseva [1]

[1] Chemistry Department, Moscow State University, Moscow 119991, Russia; pavel.kutukov@chemistry.msu.ru (P.K.); roum@inorg.chem.msu.ru (M.R.)
[2] N.N. Semenov Federal Research Center for Chemical Physics of Russian Academy of Sciences, Moscow 119991, Russia; astafiev.artyom@gmail.com (A.A.)
* Correspondence: chizhov@inorg.chem.msu.ru; Tel.: +7-495-939-5471

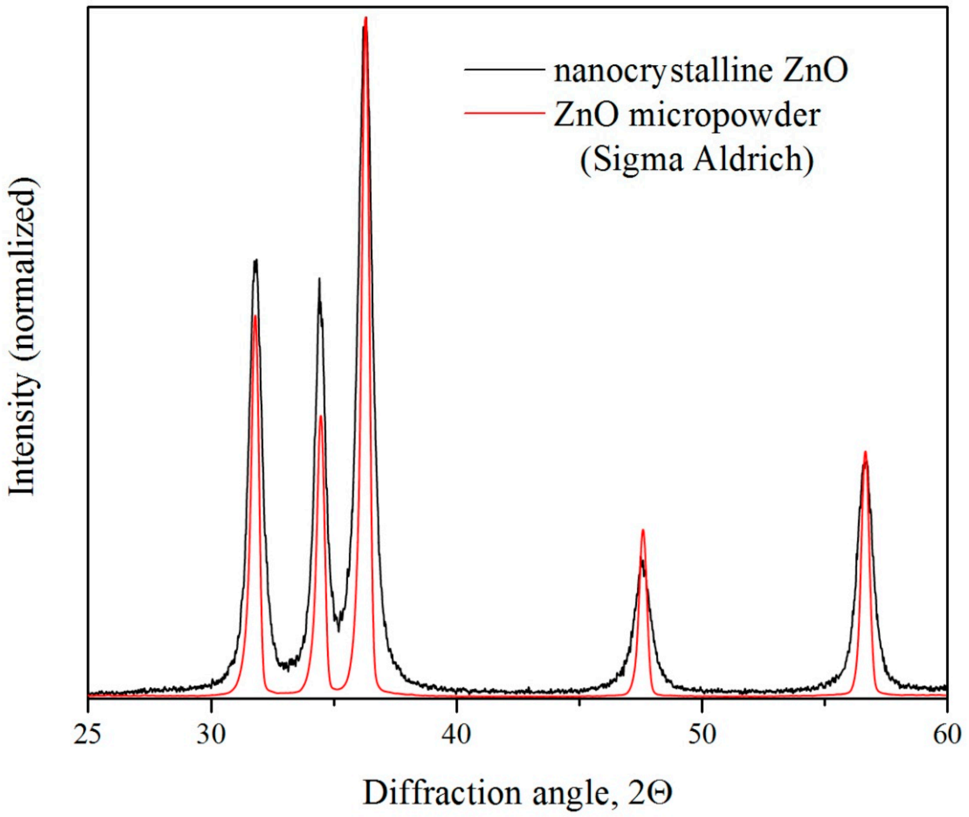

Figure S1. Comparison of XRD patterns of the synthesized nanocrystalline ZnO (annealed at 300°C) and the initial ZnO micropowder precursor (Sigma Aldrich). Intensity is normalized to (101) peak. One can see a significant difference in the width of the diffraction peaks.

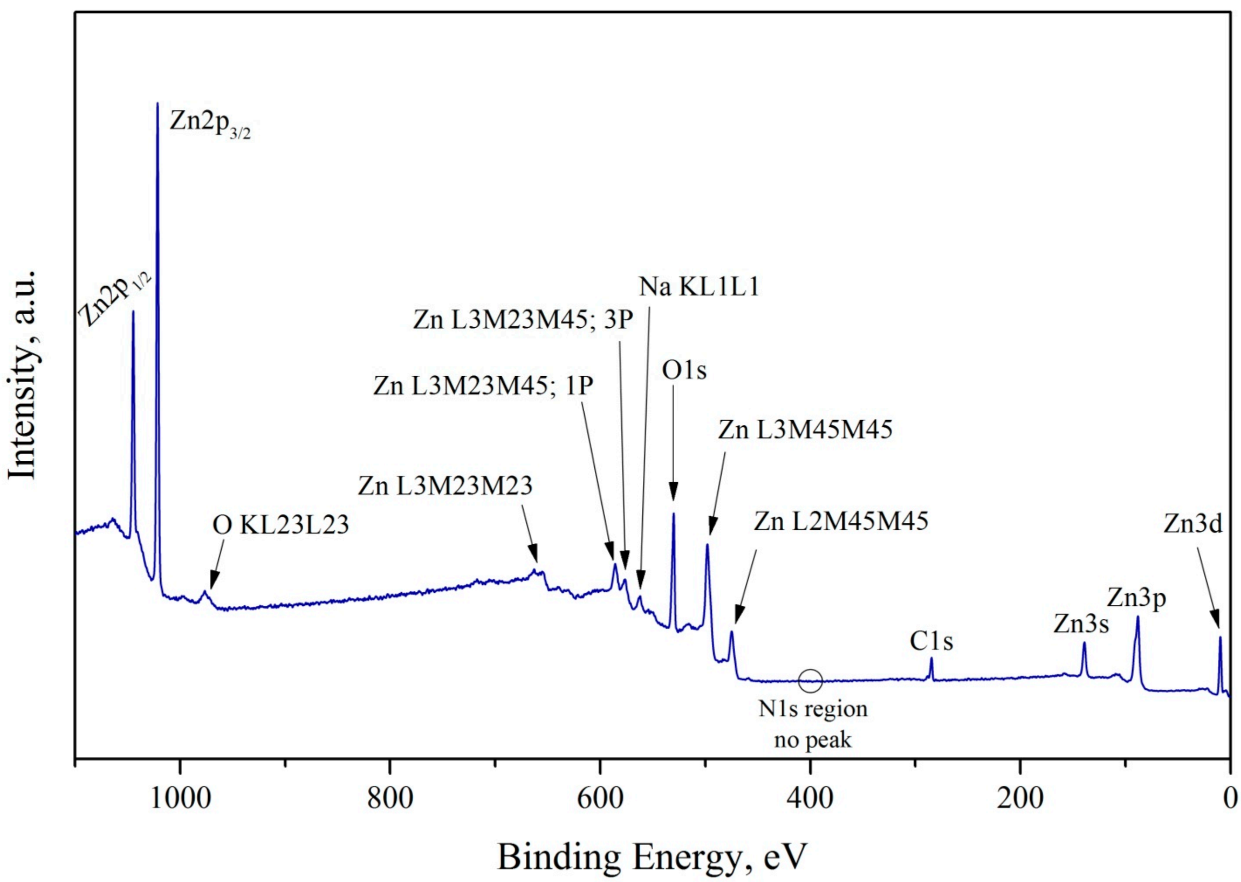

Figure S2. Survey XP spectrum of synthesized nanocrystalline ZnO. Only photoelectronic and Auger peaks of oxygen and zinc are seen in the spectrum, as well as small peaks of adventitious carbon and sodium. There is no peak in the expected N1s region.

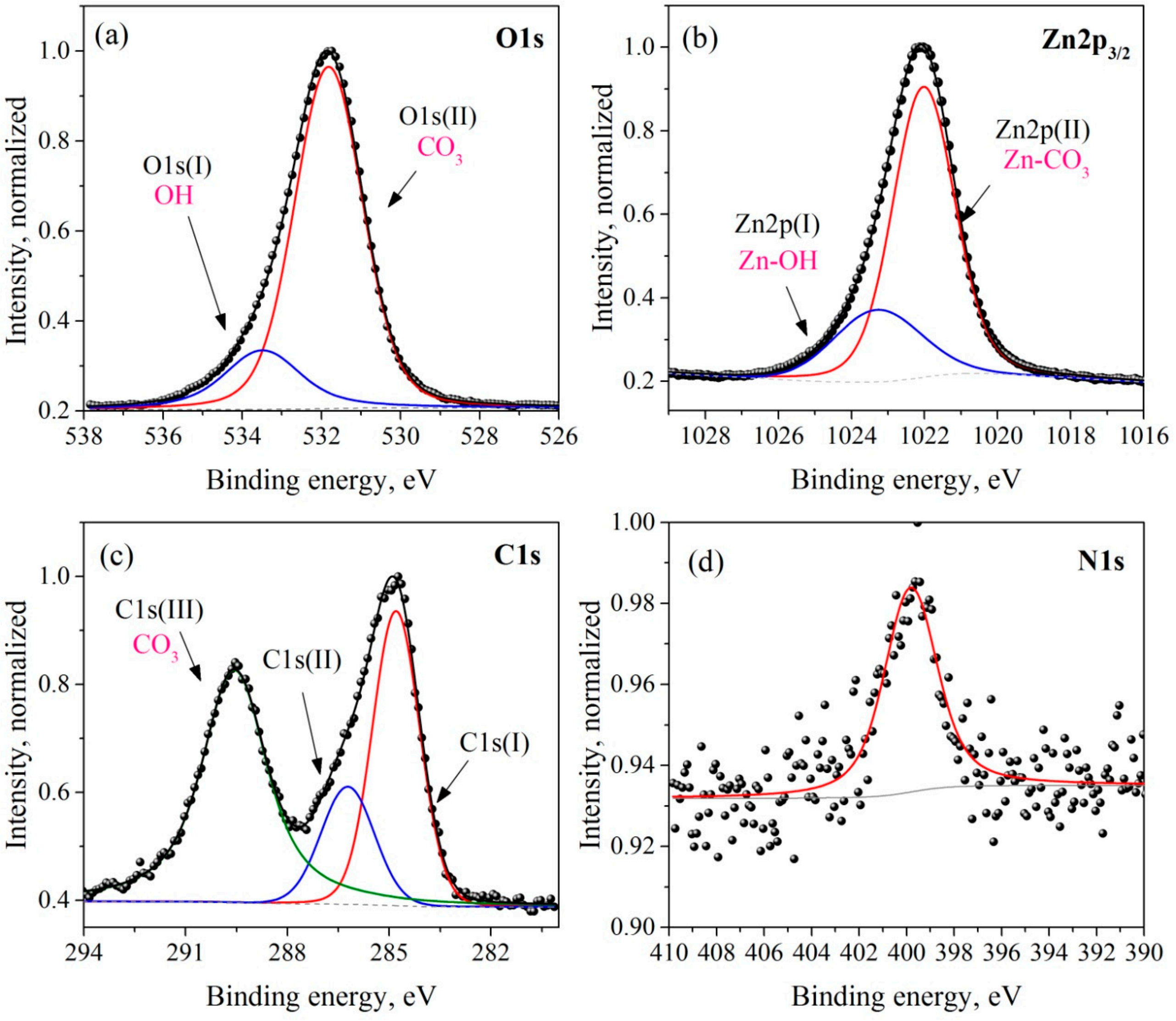

Figure S3. XP spectra of HZ nanoplates on the regions O1s (a), Zn2p3/2 (b), C1s (c) and N1s (d).

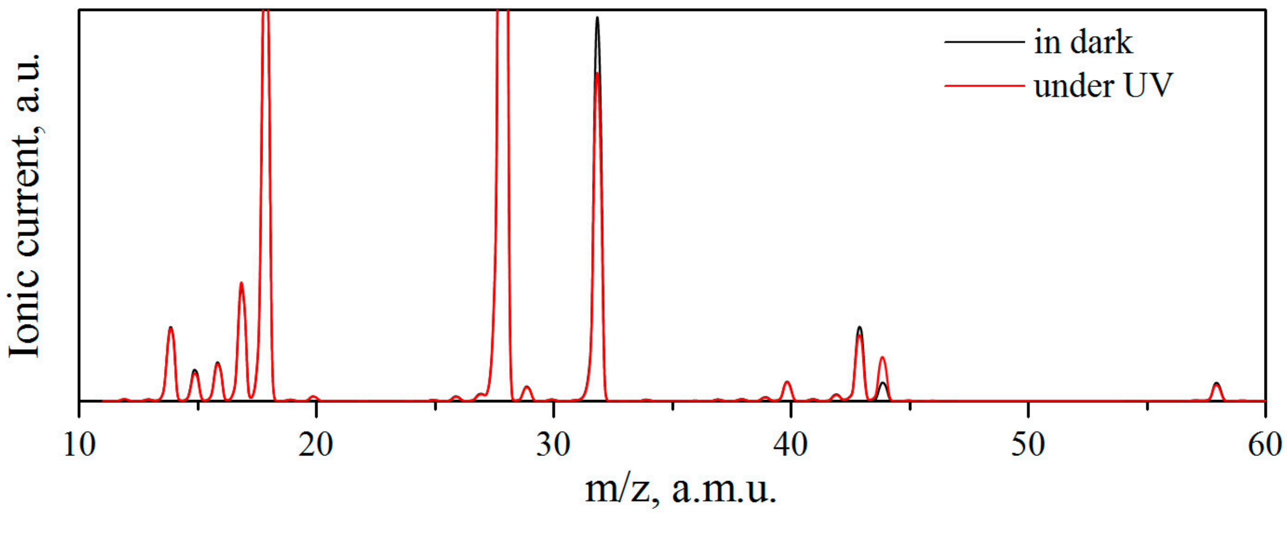

Figure S4 Full-scan mass spectra in the range of 10-60 a.m.u. of the carrier gas passed over the reference ZnO sample in the dark and during UV irradiation.

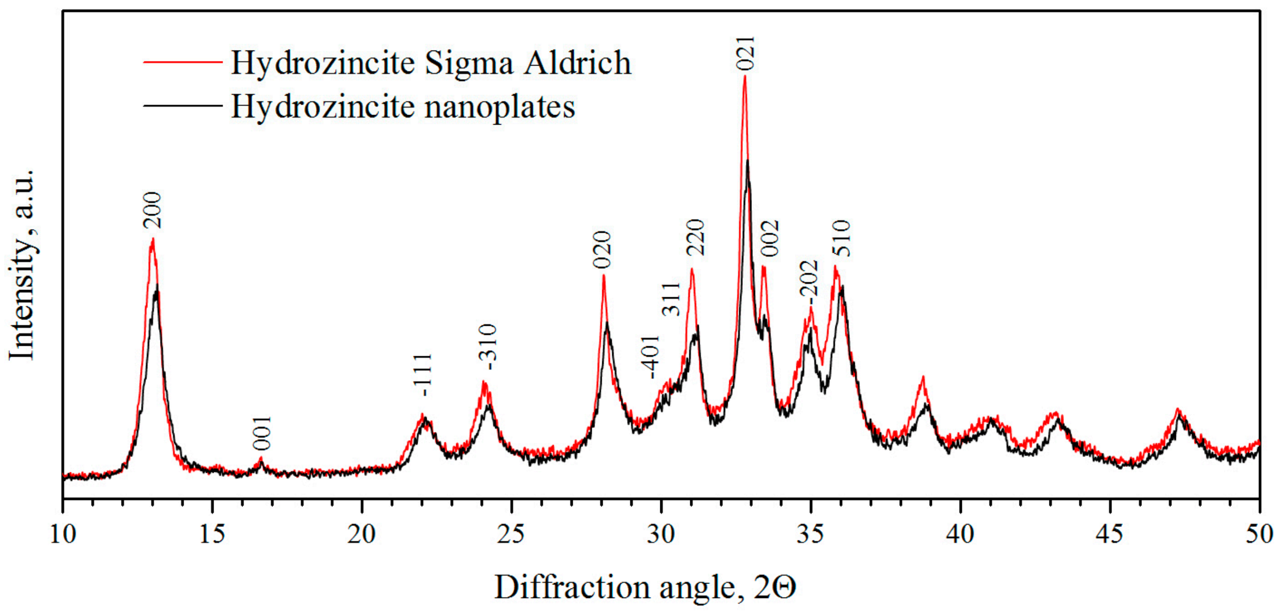

Figure S5. Comparison of XRD patterns of commercial hydrozincite powder and synthesized hydrozincite nanoplates

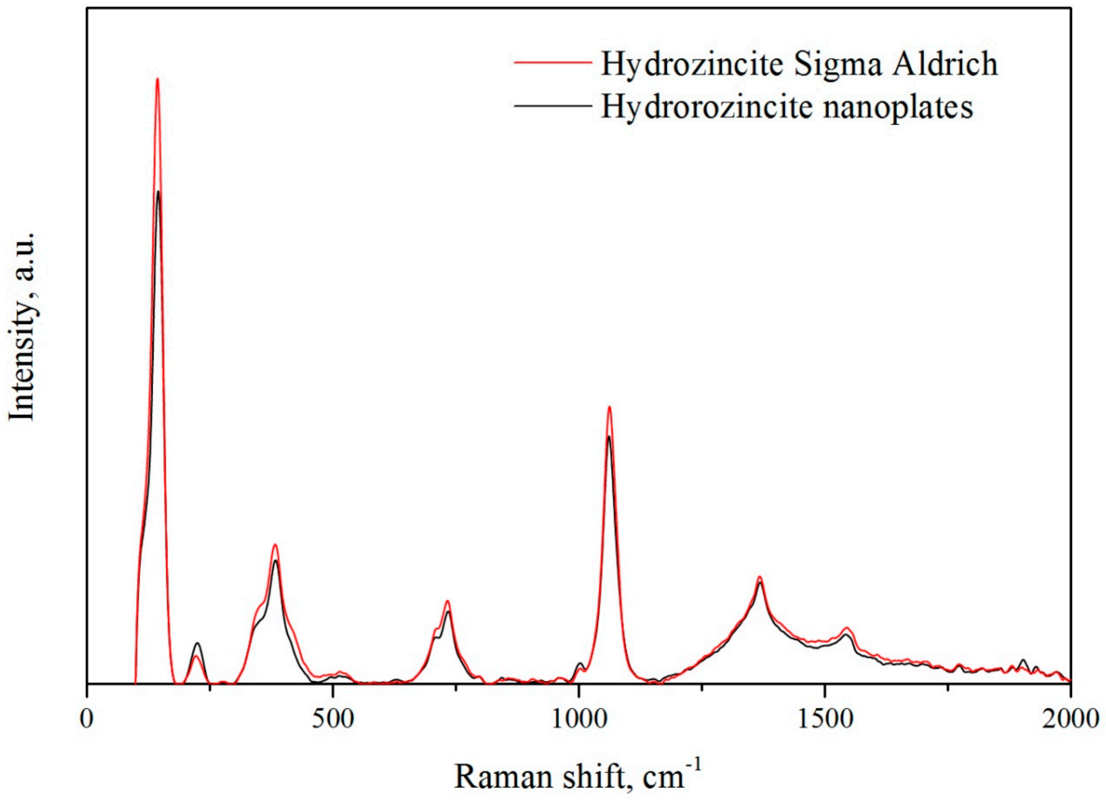

Figure S6. Comparison of Raman spectra of commercial hydrozincite powder and synthesized hydrozincite nanoplates (wavelength excitation 785 nm)

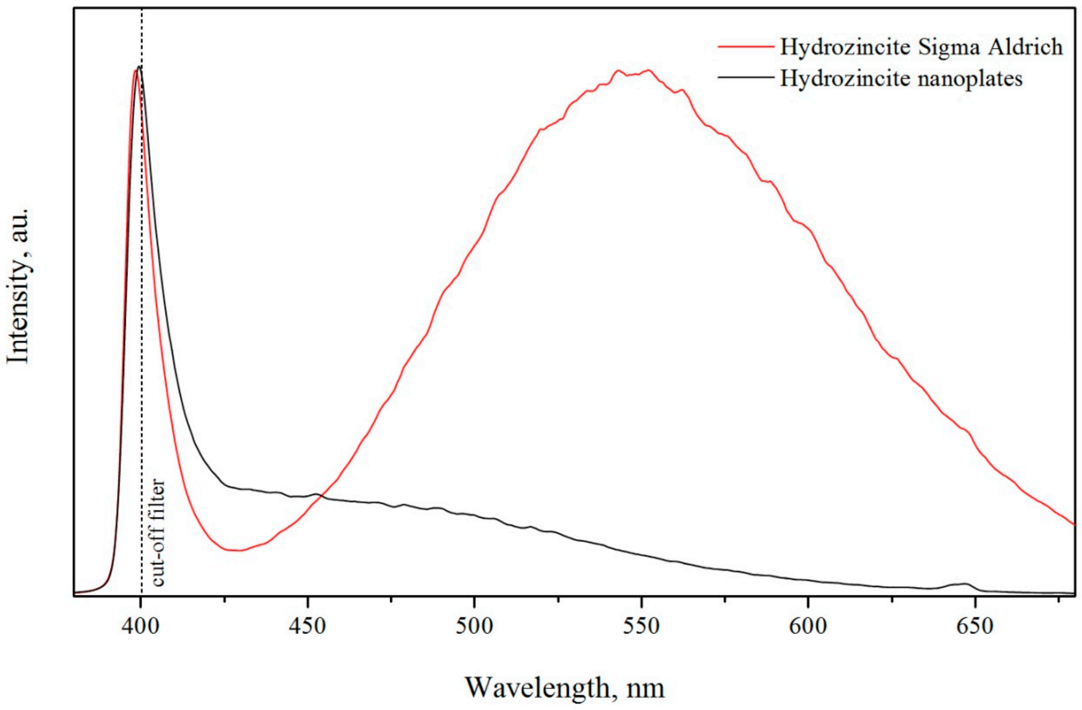

Figure S7. Comparison of PL spectra of commercial hydrozincite powder and synthesized hydrozincite nanoplates (wavelength excitation 360 nm)

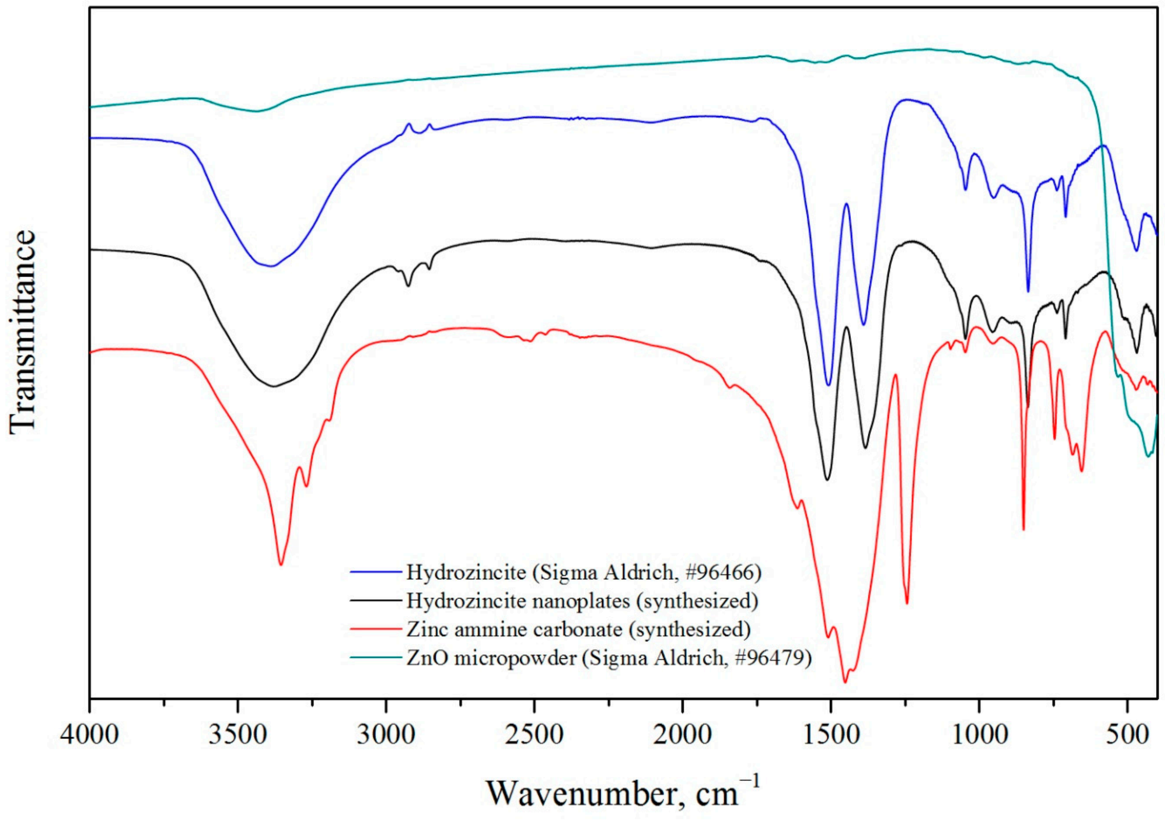

Figure S8. FTIR spectra of commercial HZ powder (Sigma-Aldrich, #96466); synthesized HZ nanoplates; synthesized zinc ammine carbonate; ZnO commercial powder (Sigma Aldrich, #96479).
